# Establishment of an open data policy for *Journal of Educational Evaluation for Health Professions*, appreciation for invited reviewers, and acknowledgement of volunteers who made audio recordings

**DOI:** 10.3352/jeehp.2017.14.37

**Published:** 2017-12-29

**Authors:** Sun Huh

**Affiliations:** Department of Parasitology and Institute of Medical Education, Hallym University College of Medicine, Chuncheon, Korea; The Catholic University of Korea, Korea

In April 2016, *Journal of Educational Evaluation for Health Professions* adopted an open data policy [1]. This step was taken to prevent “questionable research practices” and “sloppy science” [2]. The importance of open data policies was stressed at the 2014 Crossref annual meeting by Laurie Goodman of GigaScience as follows: “Open data sharing would benefit the authors as well as the general public and reduce the publication of false and irreproducible results” [3]. When an open data policy is adopted, editors can be proud of guaranteeing the replicability of results from the raw data generated from research. Therefore, this policy aims to ensure the scientific soundness of the studies published in the journal, which is the most important characteristic of the journal from the perspective of the editor. Creativity is difficult to assess because it varies according to the characteristics of the journal, such as its publication region, readers, authors, and editors. Another major contribution of data sharing to academic societies is to provide real data that can be analyzed to confirm theoretical work using simulated data. In the field of educational measurement, hypotheses are usually tested in 3 steps: (1) building a new model or method; (2) identifying the best model or method by using simulated data; and (3) applying the new model or method to real data. If the raw data of a high-stakes examinations with a sufficient number of examinees are opened to researchers in the field of educational measurement, they will be able to work more efficiently and be more motivated. It is difficult to find a journal with this kind of open data policy among the international journals dealing with education that are indexed in Medline, PubMed Central, SCOPUS, and the Web of Science Core Collection.

The authors of all articles published since April 2016 have included their raw data, which have been deposited on the journal website and PubMed Central. In March 2017, the editorial office requested Harvard Dataverse to create a journal-specific space for depositing the journal’s data. This space was created on April 11, 2017, and is available at: https://dataverse.harvard.edu/dataverse/jeehp. Therefore, starting in April 2017, all data began to be deposited in the Harvard Dataverse. Data can be deposited at this depository site at no cost. Because the Harvard Dataverse provides a digital object identifier (DOI) for each dataset, it is very convenient to present the URL for the site where data have been deposited. A simple DOI address suffices to direct a reader to the data site (for example, https://doi.org/10.7910/DVN/XSF8XW). Through the end of 2017, 111 journals have created a deposit space in the Harvard Dataverse. The number of journals registered in the Harvard Dataverse has increased year by year [4] (Fig. 1). Of those journals, this journal and the *Revista Brasileira de Educação do Campo* published by the Universidade Federal do Tocantins are the 2 journals that deal with education. I am not sure how many other journals in the field of education have announced an open data policy. There is another data repository, Figshare (https://figshare.com/), which is used by many international publishers; however, it is difficult to find information regarding open data policies on the journal level. I would guess that this journal is 1 of only 2 education journals with an open data policy at the present. Submitters to the journal understand this policy very well, and they are therefore eager to provide raw or processed data. In 2017, only 1 submitter refused to provide raw data. That article was withdrawn before the review process. I hope that many researchers access the data of the studies that we have published, replicate the results, and develop novel ideas from the data.

This year, it was possible to publish many invaluable articles due to the voluntary work of invited reviewers. I appreciate their devotion to the journal. The list of reviewers for the 2017 issue is as follows:

Su-Jin Chae, Ara Cho, Jody Chu, Kevin Chui, Chad Cook, Upreet Dhaliwal, Christian Ezeala, Jorge Riquelme Galindo, Diana Galindo, Mitra Hanani, Maureen Hardy, Jianhui He, Kyaw Ko Ko Htet, Yera Hur, Kun Hwang, Ki-Taeg Jang, Eun Young Jeon, Geum Hee Jeong, Amir Maroof Khan, Saval Khanal, Hyunjung Kim, Ki-Song Kim, Nam-Hee Kim, Kyung-Nyun Kim, Sue Kim, Shin Jeong Kim, Hyunjung Kim, Tetsuo Kimura, Oh Young Kwon, Young Hwan Lee, Kyoungsin Lee, Sang-Kyu Lee, Eunyoung Lim, Woo-Taek Lim, Christianne Micallef, Victor Mogre, Barbara O’Donell, Younjae Oh, Young Guk Park, Sonja Raaum, Jennifer Reneker, Saara Repo, Hye-Rin Roh, Dong Gi Seo, Jeong-Tak Seo, Ravi Shankar, Ramzi Sha-wahna., Jae-Hoon Shim, Sujin Shin, Dong Whan Sohn, Hohee Son, Pascal Staccini, Mohan Sunkad, Gideon Victor, Adam Wilson, Shi-geo Yamamura, Hon Yuen.

Additionally, Tom Huh has worked on a volunteer basis to make audio recordings of the abstracts.

Before closing this issue, I wish to express my regret for not accepting more articles from throughout the world. Some submissions were not accepted for a variety of reasons. I believe that all research data are worthy of being published; however, published research should fit not only the aims and scope of the journal, but also its style and formatting requirements. After verifying that submissions met those criteria, I screened them for scientific soundness. Most of the manuscripts were from medical/health educators, and I believe that their research and experiences are meaningful and helpful to other educators, not only in their country, but throughout the world. The editorial team and I are always happy when we receive well-formatted manuscripts. We have done our best to edit those manuscripts after evaluating their scientific merit. I hope to publish brilliant articles from all over the world next year as well. I close this editorial by expressing my appreciation once more to the authors, reviewers, and readers of this journal, all of whom I wish a productive and fulfilling new year.

## Figures and Tables

**Fig. 1.**
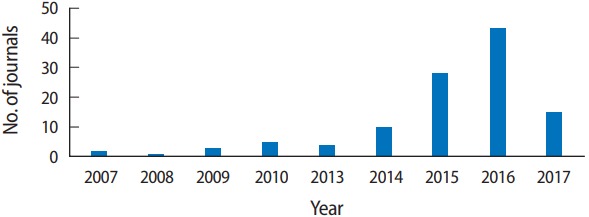
Number of journals registered in the Harvard Dataverse by year [Internet]. Cambridge (MA): Harvard Dataverse Network [cited 2017 Dec 27]. Available from: https://dataverse.harvard.edu/ [4].
